# Pathogenesis and potential reversibility of intestinal metaplasia − a milestone in gastric carcinogenesis

**DOI:** 10.2478/raon-2024-0028

**Published:** 2024-04-21

**Authors:** Jan Drnovsek, Matjaz Homan, Nina Zidar, Lojze M Smid

**Affiliations:** Department of Gastroenterology, University Medical Centre Ljubljana, Ljubljana, Slovenia; Faculty of Medicine, University of Ljubljana, Ljubljana, Slovenia; Institute of Pathology, Faculty of Medicine, University of Ljubljana, Ljubljana, Slovenia; Department of Gastroenterology, Hepatology and Nutrition, University Children’s Hospital, Ljubljana, Slovenia

**Keywords:** *Helicobacter pylori*, intestinal metaplasia, gastric cancer

## Abstract

**Background:**

Non-cardia gastric cancer remains a major cause of cancer-related mortality worldwide, despite declining incidence rates in many industrialized countries. The development of intestinal-type gastric cancer occurs through a multistep process in which normal mucosa is sequentially transformed into hyperproliferative epithelium, followed by metaplastic processes leading to carcinogenesis. Chronic infection with *Helicobacter pylori* is the primary etiological agent that causes chronic inflammation of the gastric mucosa, induces atrophic gastritis, and can lead to intestinal metaplasia and dysplasia. Both intestinal metaplasia and dysplasia are precancerous lesions, in which gastric cancer is more likely to occur. Atrophic gastritis often improves after eradication of *Helicobacter pylori*; however, the occurrence of intestinal metaplasia has been traditionally regarded as “the point of no return” in the carcinogenesis sequence. *Helicobacter pylori* eradication heals non-atrophic chronic gastritis, may lead to regression of atrophic gastritis, and reduces the risk of gastric cancer in patients with these conditions. In this article, we discuss the pathogenesis, epigenomics, and reversibility of intestinal metaplasia and briefly touch upon potential treatment strategy.

**Conclusions:**

Gastric intestinal metaplasia no longer appears to be an irreversible precancerous lesion. However, there are still many controversies regarding the improvement of intestinal metaplasia after *Helicobacter pylori* eradication.

## Introduction

The global burden of gastric cancer remains high, ranking fifth for incidence and third for cancer-related mortality worldwide. Early recognition of the disease can lead to potentially successful treatment; however, most patients are diagnosed at a late stage.^1^(1) *H. pylori* is the main risk factor for non-cardia gastric cancer development. Although most *H. pylori*-positive individuals remain asymptomatic, the infection predisposes them to the development of chronic gastritis^2^, which can be followed by the inflammation–atrophy–metaplasia–dysplasia–carcinoma sequence, known as the Correa cascade.^3^ Both chronic atrophic gastritis and intestinal metaplasia are considered precancerous conditions, as they independently confer risk for the development of dysplasia and gastric cancer.^4^

*H. pylori* infection is associated with a 3-fold increase in the lifetime risk for developing non-cardia gastric cancer, and *H. pylori* infection is believed to cause at least 75% of all gastric cancer.^5^ The eradication reduces the risk of gastric cancer in patients with non-atrophic and atrophic gastritis and effectively heals non-atrophic chronic gastritis. It may also lead to the regression of atrophic gastritis.^6^ On the other hand, short-term cancer risk in patients with established intestinal metaplasia does not seem to change significantly with *H. pylori* eradication^7,8^, and intestinal metaplasia has thus been considered irreversible. This concept has been challenged in recent years by studies with longer follow up, in which regression of intestinal metaplasia has been observed after *H. pylori* eradication.^9,10^ This short review summarizes the role of *H. pylori* in intestinal metaplasia and non-cardia gastric cancer, reviews gastric intestinal metaplasia pathogenesis, and briefly discusses evidence regarding its reversibility.

The following keywords and MeSH terms were used for online searches: [(gastric) AND (metaplasia) OR (intestinal) AND ((regression) OR (reversibility) OR (reversible))]. Reference lists of suitable studies and related previous review articles were reviewed manually to increase search yield and identify other related studies. All searches were restricted to original studies published in the English language.

### Helicobacter pylori infection and intestinal metaplasia

*H. pylori*, a microaerophilic, spiral-shaped, Gram-negative bacterium, colonizes the gastric epithelium in over half of the adult population worldwide. Its prevalence varies widely, ranging from 30% in industrialized regions to 90% in developing countries and Eastern Asia.^11,12^ *H. pylori* stands as the most potent single risk factor for non-cardia gastric cancers, including adenocarcinoma and lymphoma^13^ and was classified as a class I carcinogen by the International Agency for Research on Cancer (IARC) and the World Health Organization (WHO) in 1994. Gastric adenocarcinoma is generally divided into two main histological subtypes: diffuse and intestinal, and *H. pylori* contributes to the risk of both.^14^

Diffuse-type gastric adenocarcinomas, characterized by poorly differentiated infiltrating neoplastic cells without a clear glandular structure, predominantly occur in younger patients. Their development does not require long-standing chronic inflammation, and *H. pylori*’s exact role in this subtype remains unclear. Diffuse-type cancer is associated with interference in cell adhesion, polarity, and proliferation, all caused by *H. pylori* infection, leading to the cleavage of E-cadherin, abnormal intracellular accumulation of β-catenin, TP53 mutations, and reduced p27 protein expression.^15^ On the other hand, intestinal-type gastric adenocarcinoma emerges later in life and consists of irregular glandular structures formed by well-differentiated cancer cells. This type represents the terminal phase of the chronic inflammation-atrophy-metaplasia-dysplasia-carcinoma sequence, initiated by *H. pylori*-induced gastritis.^16^ Atrophic gastritis and gastric intestinal metaplasia, which evolve over decades of chronic infection, are thus established pre-neoplastic lesions for intestinal-type gastric adenocarcinoma.^17^ This sequence allows for the possibility of primary prevention strategies involving either population-based or targeted screening to identify patients with precancerous lesions who may need subsequent surveillance.^18^

*H. pylori* utilizes urease activity to neutralize the acidic conditions in the host stomach at the infection’s onset. The bacterium’s flagella-mediated motility enables movement toward host gastric epithelium cells. This movement, followed by interactions between bacterial adhesins and host cell receptors, facilitates successful colonization and persistent infection. Some strains of *H. pylori* release effector proteins and toxins, such as cytotoxin-associated gene A (CagA) and vacuolating cytotoxin A (VacA), which can damage host tissue.^19^ A direct correlation exists between the number of virulence factors in an *H. pylori* strain and the frequency of associated advanced gastric mucosa pathology.^20^ However, the characterization of *H. pylori* virulence genes’ individual roles is complex due to the interaction of methodological^21^, bacterial, and host factors^19^, often leading to conflicting results and interpretations.

Intrabacterial urease activity is required for *H. pylori* acid resistance, and this activity is regulated by the proton-gated urea channel UreI, which permits urea entry only under acidic conditions and thus prevents lethal alkalization during times of relative neutrality. The urease gene cluster is composed of seven genes, including catalytic subunits (*ureA/B*), an acid-gated urea channel (*ureI*), and accessory assembly proteins (*ureE-H*).^22^ Urease can also protect against host innate immune response by modulation of phagosome pH following phagocytosis and promotion of *H. pylori* survival inside megasomes.^23^

Flagella-mediated motility is essential for colonization of the gastric mucosa by *H. pylori*. Loss of any component of the motility and chemotaxis systems abolishes the ability of *H. pylori* to infect the stomach and establish colonization.^24,25,26^ Infection with *H. pylori* that exhibits higher motility may show enhanced bacterial density, triggering a more pronounced inflammatory response in the upper stomach, and can thus be associated with severe pathological outcomes.^27^ The flagellar filament consists of two flagellins (FlaA and FlaB) encoded by *flaA* and *flaB*.^28^ FIaA elicits host antibody response and can be used as a marker of *H. pylori* infection; host anti-FlaA titer correlates with *H. pylori* colonization density^29^ and the presence of gastric intestinal metaplasia.^30^

The interaction of bacterial adhesins with host cellular receptors protects *H. pylori* from displacement by the forces generated by peristalsis. This bacterial adherence plays an important role in both the initial colonization and long-term persistence of *H. pylori* in the human gastric mucosa^31^ and is necessary for the tight adherence of the bacteria to gastric epithelial cells, which facilitates subsequent delivery of bacterial toxins.^32^ The *H. pylori* genome encodes a variety of outer membrane proteins (OMPs); several OMPs have been described in detail to date, with most studies focusing on *babA2*, *oipA*, *homB*, and *sabA* genes.^19^ BabA is one of the most studied *H. pylori* adhesins. BabA is capable of binding to Lewis b and related ABO antigens on gastric epithelial cells^33^, which may play a crucial role in the development of *H. pylori* related gastric pathology such as severe gastritis, peptic ulcers, and gastric adenocarcinoma.^21,34^ *BabA* positive strains appear to be associated with worse clinical outcomes in several studies^35,36,37^, while another study found no correlation between the presence of *babA2* positive strains and atrophy or intestinal metaplasia.^21^ *HomB* may be strongly associated with gastric cancer in certain populations^38^ and display little measurable virulence in others.^39^

Attachment of *cagA*-positive *H. pylori* to host gastric epithelial cells initiates and facilitates the formation of the bacterial type IV secretion system, involved in the delivery of CagA into host epithelial cells.^32^ The translocated CagA protein localizes to the inner surface of the plasma membrane via interactions with phosphatidylserine and subsequently undergoes tyrosine phosphorylation by the Src family protein tyrosine kinase. However, once injected into the cytoplasm, CagA can alter host cell signaling in both a phosphorylation-dependent and phosphorylation-independent manner. The phosphorylated CagA binds to the phosphatase SHP-2, forming CagA-SPH-2 complex, and affects the adhesion, spreading, and migration of the cell.^40,41^ CagA can also affect the host cell in a phosphorylation-independent manner by stimulating the gastric epithelium cells to secrete IL-8, which strongly affects the level of mucosal inflammation.^42,43^

The CagA-SHP-2 complex is predominantly located in atrophic gastric mucosa and is associated with the transition to atrophic gastritis and possibly intestinal metaplasia.^41^ Deregulation of the SHP-2 role by CagA is functionally similar to the effect of the gain-of-function mutation of the SHP-2 gene observed in other human malignancies.^44^ CagA interference with intracellular signaling may thus lead to deregulation of cellular growth, apoptosis, and elevated cell motility. This can result in increased cell turnover, which in turn leads to the accumulation of further genetic changes favoring neoplastic cell transformation.^45^ Unsurprisingly, infection with *cagA*-positive strains markedly increases the risk of gastric cancer.^46^ *CagA*-positive strains are responsible for 60% of *H. pylori* infections in individuals worldwide.^47,48,49^ Strains isolated in East Asian countries such as Japan, China, and Korea are almost all *CagA*-positive.^50^ Furthermore, CagA protein can be divided into the Western-type CagA and East Asian-type CagA. The affinity of the East Asian-type CagA to SHP-2 is significantly higher than that of the Western-type CagA and is more likely to be associated with gastric cancer.^40,51^

VacA, another key toxin involved in *H. pylori* pathogenesis, binds to host epithelial cells after secretion from the bacteria. It is then internalized and causes the accumulation of large intracellular vesicles (vacuolation), interferes with mitochondria, and causes apoptosis of host cells.^52^ VacA also appears to disrupt the balance of cell proliferation and death by affecting genes that regulate the cell cycle.^53^ *H. pylori* strains producing VacA differ in the potency of cytotoxin, in both its activity (allele s1 is more active than s1) and binding (allele m1 is more effective than m2).^54^ A meta-analysis of 33 studies (1,446 cases and 2,697 controls in total) confirmed the correlation between the *vacA s1* genotype and the risk of atrophic gastritis, intestinal metaplasia, and gastric cancer. The *vacA m1* genotype was associated with intestinal metaplasia and gastric cancer but did not significantly correlate with atrophic gastritis.^55^

## Pathogenesis of gastric intestinal metaplasia

**FIGURE 1.**
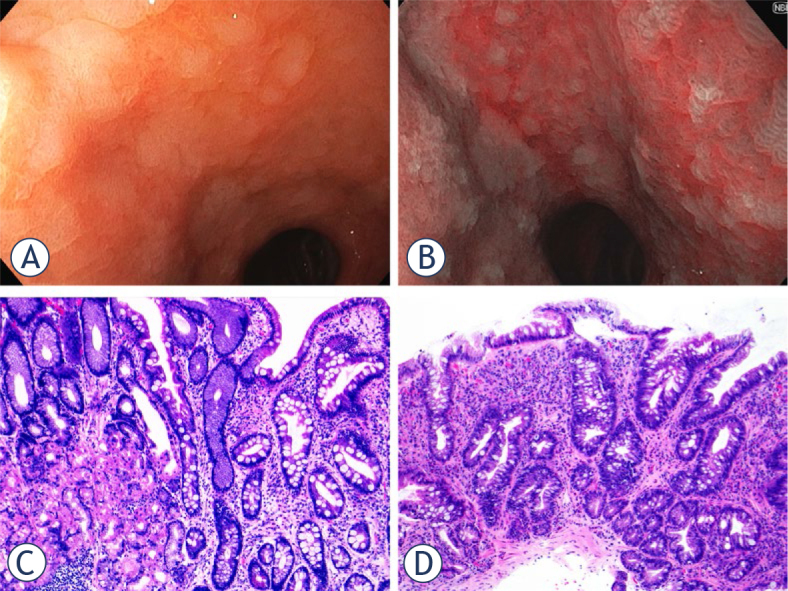
Gastric intestinal metaplasia, endoscopic **(A, B)** and histological **(C, D)** appearance. Gastric intestinal metaplasia is endoscopically characterized by the presence of grey-white velvety or slightly nodular elevated patches, which are clearly demarcated against the surrounding pink gastric mucosa, as illustrated in image A of antral gastric mucosa under white light. Narrow band imaging (NBI, depicted in image **B**) further enhances the visualization of mucosal and vascular patterns by employing optical filters to narrow the bandwidth of light. This technique offers superior contrast compared to white light endoscopy, thereby improving the detection of metaplastic transformation. Histologically, gastric intestinal metaplasia can be classified into either complete (as seen in image **C**) or incomplete types (as shown in image **D**). Image **C** demonstrates preserved oxyntic mucosa (on the left) adjacent to intestinal metaplasia of the complete type, which features enterocytes with a well-defined brush border, alongside well-formed goblet cells and Paneth cells. In contrast, image **D** illustrates the intestinal metaplasia of the gastric mucosa of the incomplete type, characterized by goblet cells of variable size and intervening mucin-secreting columnar cells that lack a brush border (both images are hematoxylin and eosin-stained, original magnification 10x).

Gastric intestinal metaplasia is defined as the replacement of normal gastric epithelium in the antral or oxyntic mucosa with intestinal epithelium, consisting of intestinal cell types including Paneth, goblet, and absorptive cells.^56^ These metaplastic glands are characterized by modification of the surrounding stroma and by reorganization of the crypts, with displacement of the proliferative zone from the neck region to the base of the crypts.^57^ Intestinal metaplasia can be classified as either limited (when confined to one anatomical region) or extensive, if two gastric regions are involved (Figure 1).

Complete intestinal metaplasia is characterized by small intestinal-type mucosa with mature absorptive cells, and a brush border, with a notable loss of gastric mucin markers (MUC1, MUC5AC, MUC6) and an acquisition of the intestinal mucin MUC2. On the other hand, incomplete intestinal metaplasia is characterized by columnar “intermediate” cells at various differentiation stages, irregular mucin droplets, and a lack of a brush border, while still maintaining gastric mucin markers alongside the presence of intestinal mucin MUC2.^58,59^ Earlier gastric metaplasia classifications relied on traditional mucin staining methods (such as periodic acid-Schiff, Alcian blue, and high iron diamine) and cell morphology. This methodology defined three intestinal metaplasia grades: Type I, which encompasses absorptive cells, Paneth cells, and goblet cells that secrete sialomucins; Type II, consisting of goblet and columnar cells secreting sialomucins; and Type III, involving goblet and columnar cells secreting sulfomucins. Presently, Type I aligns with the complete type, while Types II and III correspond to the incomplete type in the contemporary classification.^58^

**FIGURE 2.**
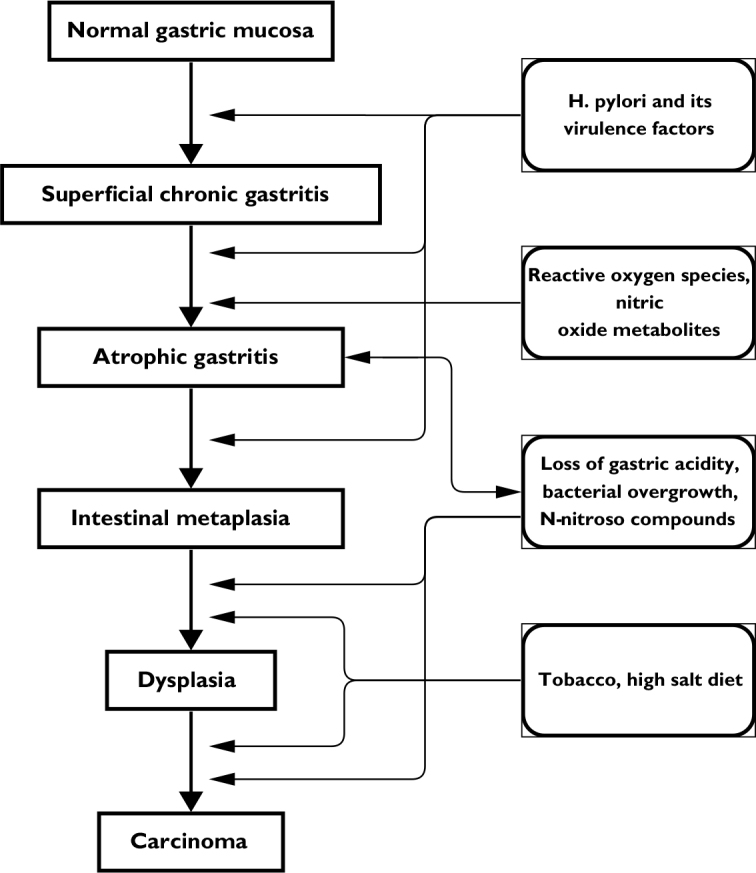
Pathogenesis of intestinal metaplasia and gastric adenocarcinoma – the Corea cascade. This stepwise process starts with chronic gastritis triggered by *H. pylori* infection. The likelihood of developing gastric cancer is higher in individuals infected with virulent strains of *H. pylori*, unhealthy diets (rich in salt and smoked foods), low iron levels, and harmful lifestyle choices, including smoking. Persistent inflammation results in the damage and eventual loss of acid-producing parietal cells, causing reduced stomach acidity (hypochlorhydria) and eventually no stomach acid production (achlorhydria). This reduction in acidity allows for the colonization of the stomach by detrimental, pro-inflammatory microbiota. These bacteria can produce genotoxic and pro-inflammatory metabolites and carcinogens, directly contributing to the transformation of stomach epithelial cells into malignant cells.

The Correa cascade is a widely accepted model of the pathogenesis of gastric cancer (Figure 2).^3^ This cascade commences with the emergence of chronic mucosal inflammation, mediated by polymorphonuclear and mononuclear cells. It evolves through a multifactorial process, steered by various factors including *H. pylori*, host genetics, environmental elements, and diet, propelling further alterations in the gastric mucosa towards atrophy, metaplasia, and ultimately, cancer.^60,61,62^

Annually, an estimated 0.1%, 0.25%, 0.6%, and 6% of Western patients with atrophic gastritis, intestinal metaplasia, and mild-to-moderate or severe dysplasia, respectively, progress to gastric cancer.^62^ In contrast, East Asian populations demonstrate a higher risk, with about 1.8%, 10%, and 73% of patients with atrophic gastritis, intestinal metaplasia, and dysplasia, respectively, progressing to gastric cancer each year.^63^ Patients with incomplete intestinal metaplasia encounter a 3.3-fold higher relative risk of developing gastric cancer compared to those with complete intestinal metaplasia. Furthermore, extensive intestinal metaplasia is linked with a 2.1-fold higher relative risk of progression compared to limited gastric metaplasia.^64,65^

Host factors that are associated with higher risk for non-cardia gastric cancer are similar to risk factors for development of intestinal metaplasia (Table 1) and include advanced age, male sex, family history, and smoking. More than two thirds of all gastric cancers are diagnosed after the age of 55, and roughly two thirds of non-cardia cancers are found in male patients.^66^ The reason for the latter observation is most likely multifactorial. The difference can be partly attributed to smoking (which is more prevalent in men) and partly to the protective role of estrogen, since increased fertility and late menopause both reduce the risk of gastric cancer in women.^67^ Individuals with blood type A have a 20% higher chance of developing gastric cancer when compared to other blood types, according to a prospective blood donor cohort study.^68^

**TABLE 1.** Patients’ related predictive risk factors for gastric intestinal metaplasia

**Risk Factor**	**Odds ratio (OD)**	**Key findings**	**References**
**Race**			
**White**	1	Hispanic and Asian patients have an increased risk for GIM	Tan MC *et al.* (2022)^94^
**Asian**	2.83–3		Akpoigbe K *et al.* (2022)^95^
**Hispanic**	2.10–5.6		
**Age (> 50 years)**	1.5–2.03	Risk increases with age, possibly due to accumulated exposure to risk factors.	Aumpan N *et al.* (2021)^96^
			Tan MC *et al.* (2020)^97^
**Male gender**	1.55–2.09	Probably due to genetics and exposure to other risk factors	Aumpan N *et al.* (2020)^98^
			Leung WK *et al.* (2005)^99^
**Chronic gastritis**	3.68–5.76	Chronic inflammation is leads to IM.	Yoo YE *et al.* (2013)^100^
			Tatsuta M *et al.* (1993)^101^
***H. pylori* infection**	2.47–3.65	Strong correlation with IM, especially with *CagA* positive strains.	Aumpan N *et al.* (2021)^96^
			Nguyen T *et al.* (2021)^102^
**Family history of gastric cancer**	1.5–3.8	Patients with a first-degree relative with gastric cancer have an increased risk of neoplastic progression	Nieuwenburg SAV *et al.* (2021)^103^
			Reddy KM *et al.* (2006)^104^
**Alcohol consumption**	1.27–1.54	Alcohol intake was independently associated with increased risk of developing AG and IM	Holmes HM *et al.* (2021)^105^
			Kim K *et al.* (2020)^106^
**Tobacco smoking**	1.54–2.75	Tobacco smoking is a risk factor for gastric IM.	Morais S *et al.* (2014)^107^
			Thrift AP *et al.* (2022)^108^
**Blood group A**	1.39–1.42	Blood group A is associated with higher risk of GIM	Mao Y *et al.* (2019)^109^
			Rizatto C *et al.* (2013)^110^
**Bile reflux**	unknown	Bile acids not only interefere with gastric mucosa but also regulate multiple carcinogenic pathways	Wang M *et al.* (2023)^111^
			Yu J *et al.* (2019)^112^
**Salt consumption**	0.37–1.53	Salt intake may increase progression to advanced gastric precancerous lesions	Dias-Neto M *et al*. (2010)^113^
			Song JH *et al.* (2017)^114^
**Industrially processed food**	unknown	Dietary exposure to *N*-nitroso–containing compounds has been shown to increase the promotion of gastric carcinogenesis	Wiseman M (2008)^115^
			Jencks DS *et al.* (2018)^116^

Ethnicity also plays an important role in gastric cancer risk. The incidence of non-cardia gastric cancer in individuals of African-American, East Asian, or Pacific Islander descent is almost twice that observed in Caucasians.^69^ A similar pattern was seen in the analysis of intestinal metaplasia prevalence. A study that reviewed 800,000 gastric biopsies taken in the United States showed 20% prevalence of gastric metaplasia in people of East Asian descent, 12% prevalence in Hispanics, and 8% in all other ethnic backgrounds.^62^

Tobacco smoking is the second most important environmental factor in gastric cancer pathogenesis, accounting for 11% of all cases.^70^ Tobacco use increases the risk of intestinal metaplasia and doubles the risk of its progression to dysplasia, according to a large Chinese population-based study.^71^

Bile acid reflux into the gastric lumen produces repetitive gastric mucosal injury, which predisposes patients to intestinal metaplasia and gastric cancer in *H. pylori*-positive patients.^72^ Bile acids increase the expression of CDX2, an intestinal-specific transcription factor that directs and maintains intestinal differentiation in gastric mucosa^73^, and indirectly damage cellular DNA by induction of oxidative stress and production of reactive oxygen species^74^, which promote intestinal metaplasia and the further accumulation of mutations, leading to increased cancer risk.

The role of diet (being an obvious potential factor in gastric disorders) has been extensively studied in gastric cancer pathogenesis. High salt consumption is associated with increased risk of *H. pylori* infection and upregulation of *cagA* expression.^75,76^ Dietary use of processed or preserved meat using smoke or salt is positively and dose-dependently associated with non-cardia gastric cancer.^77^ Nitrite and nitrate additives form N-nitroso carcinogenic compounds when they combine with amino acids.

Similar carcinogens are formed by ingestion of haem (and meat) in the human gastrointestinal tract.^78^ Vegetables and fruits in the diet have a protective role^79^, and folic acid supplementation has been shown to reduce *H. pylori* related gastric inflammation and dysplasia in murine models.^80^

## Reversibility of intestinal metaplasia

Large prospective trials of *H. pylori* eradication for non-cardia gastric cancer prevention failed to show a reduction in gastric cancer incidence after eradication in a subpopulation of patients with pre-existing gastric intestinal metaplasia or extensive atrophic gastritis.^7,81^ Intestinal metaplasia has thus been considered irreversible, and its occurrence is considered to be the histological point of no return in the carcinogenic cascade.

These assumptions appeared to be confirmed by prospective studies designed to evaluate the effect of *H. pylori* eradication on intestinal metaplasia and atrophic gastritis in eradicated subjects. A marked regression of histologic changes associated with acute and chronic gastritis was observed after eradication in one of these studies; however, the level of mucosal atrophy and intestinal metaplasia remained unchanged one year after *H. pylori* eradication.^82^ Similar results with no regression in intestinal metaplasia were reported in a more recent detailed histological analysis of 88 antral biopsies taken in patients with intestinal metaplasia prior to and several months after *H. pylori* eradication.^83^ Several other smaller studies, all with short intervals of observation, reported similar results.^84,85^ On the other hand, a number of prospective studies with longer observation intervals report the partial regression of intestinal metaplasia.^10,86–87^ Hwang *et al*. postulated that the reason for this apparent discrepancy might stem simply from the slow pace of the process under observation.^10^ The partial reversibility of intestinal metaplasia after *H. pylori* eradication is also indirectly supported by a meta-analysis that confirmed reduced gastric cancer incidence in all levels of baseline risk, including patients with gastric metaplasia.^88^ Another recent meta-analysis directly addressed the natural course of intestinal metaplasia. Its regression was observed in 32%, and its persistence in 43%, of 20 relevant studies.^89^

A recent study of genomic and epigenomic profiling of intestinal metaplasia by Huang *et al*.^90^ also addressed the regression of intestinal metaplasia. Eighty-two eradicated patients with intestinal metaplasia were included in an assessment of correlates between molecular features and clinical outcome. At the end of surveillance period, 6 patients had developed dysplasia or cancer, 61 showed no change, and regression of intestinal metaplasia was observed in 15 patients. The level of DNA methylation changes correlated with the tendency to progress and was highest among progressors, intermediate in the stable group, and low in patients with intestinal metaplasia regression. Furthermore, *H. pylori* burden correlated with DNA methylation levels only in the intermediate group, but not in the methylation-high group, which could explain the failure of *H. pylori* eradication to stabilize or reverse intestinal metaplasia in these patients. Levels of aberrant DNA methylation could thus indicate the point of no return within the scope of intestinal metaplasia.

Folate is water soluble vitamin that acts a as a methyl group donor in DNA methylation and plays an important role in epigenetic regulation.^91^ Folic acid (FA) supplementation has been shown to reduce the risk of gastric cancer in 7-year prospective randomized trial of 216 patients with chronic atrophic gastritis.^92^ All 5 observed gastric cancer cases occurred outside the group of FA-treated patients. Furthermore, the use of FA for 12 months was associated with more frequent reversal of both, atrophy and intestinal metaplasia in comparison to patients receiving placebo. These observations were confirmed by recent meta-analysis of the role of FA supplementation in reversal of gastric precancerous conditions.^93^ Daily doses of 20–30 mg of FA in the duration of 3–6 months were associated with significant reversal of both, atrophic gastritis and intestinal metaplasia (RR: 1.77, 95% CI: 1.32–2.37).^93^

## Conclusions

The long-held belief that intestinal metaplasia of the gastric mucosa represents an irreversible precursor to cancer has increasingly been questioned in recent years. The concept of a ‘point of no return’ in the progression toward gastric cancer is now understood to be more complex than histomorphological changes alone. Consequently, the histological subtypes of gastric intestinal metaplasia must be considered during the planning of patient surveillance due to their varying potential for neoplastic transformation. Additionally, epigenomic alterations and molecular profiling could prove valuable in identifying the pro-carcinogenic transformation of intestinal metaplasia in patients without established risk factors. The eradication of *H. pylori* remains a critical step towards the potential reversibility of intestinal metaplasia; however, identifying patients at high risk of progression to cancer continues to be essential. The question of intestinal metaplasia progression despite *H. pylori* eradication could be addressed by examining changes in DNA methylation levels. Furthermore, non-*H. pylori* related host risk factors in the pathogenesis of gastric cancer are under thorough investigation. Significant challenges remain, such as accurately quantifying these factors and determining their exposure duration to assess their actual impact on intestinal metaplasia progression accurately. Recent studies highlighting the role of bile acids, N-nitroso–containing compounds, and deficiencies in vitamin C and folate have shown promise, yet their clinical relevance remains to be fully elucidated. An enduring unresolved issue is the long-term monitoring of these individuals, where the patchy nature of intestinal metaplasia could lead to sampling errors and potentially incorrect assessments of intestinal metaplasia reversibility.
